# Early weaning increases anxiety via brain-derived neurotrophic factor signaling in the mouse prefrontal cortex

**DOI:** 10.1038/s41598-019-40530-9

**Published:** 2019-03-08

**Authors:** Takefumi Kikusui, Natsumi Kanbara, Mariya Ozaki, Nozomi Hirayama, Kumiko Ida, Mika Tokita, Naho Tanabe, Kuriko Mitsuyama, Hatsuki Abe, Miki Yoshida, Miho Nagasawa, Kazutaka Mogi

**Affiliations:** Companion Animal Research, School of Veterinary Medicine, Azabu University, Sagamihara, 252-5201 Japan

## Abstract

Deprivation of maternal care during early development markedly affects emotional development, but the underlying neuromolecular mechanisms are not fully understood. In a mouse model of disrupted mother-infant relationship, early weaning causes long-term impacts on pups to exhibit increased corticosterone secretion, anxiety, and stress responses in their adulthood. Revealing the molecular mechanisms behind it would beneficial to ameliorating mental problems caused by abuse in childhood. We report that normalizing circulating corticosterone in early-weaned mice, either in adulthood or soon after weaning, ameliorated anxiety levels assessed in the plus maze test. Administering a glucocorticoid receptor antagonist into the prefrontal cortex (PFC) reversed the effects of early weaning, whereas administering corticosterone increased anxiety levels, suggesting that the PFC is corticosterone’s target brain region. In the PFCs of early-weaned mice, we observed prolonged reductions in the expression of brain-derived neurotrophic factor (BDNF) and associated mRNAs. Anxiety in early-weaned mice was ameliorated by pretreatment with BDNF or a BDNF receptor agonist. In summary, early weaning increased anxiety levels by modulating glucocorticoid and BDNF signaling in the PFC.

## Introduction

Mammalian infants heavily depend on their mothers, and mother-infant interactions greatly influence neurobehavioral development. Human children who experience maternal deprivation or neglect are at greater risk for future psychiatric illnesses^1,2^. In rodent studies, daily maternal separation in the first postnatal fortnight causes serious consequences later in life, including increased anxiety and enhanced neuroendocrine stress responses^3–5^. Early separation from the dam in late-suckling rats and mice also negatively affects neurobehavioral development^6,7^. Early weaning consistently produces adult mice with increased anxiety-like behaviors and aggression^8–10^, delayed conditioned-fear extinction^11^, and decreased empathy^12^. Early-weaned mice also exhibit heightened hypothalamic–pituitary–adrenal axis (HPA) activity, a marker of physiological stress, in response to mild stressors^13^ and novel environments^14^. These results indicate that the developing brain in late suckling is vulnerable to stress and shaped by the social environment, that these juvenile social experiences are encoded in the brain, and that these experiences have enduring behavioral effects. We hypothesize that immature mammalian brains are highly plastic and encode social experiences to guide future behavior.

Juvenile social experiences are encoded in the brain via epigenetic modifications that are believed to regulate behavioral changes. We have demonstrated that early weaning causes precocious myelination in the basolateral amygdala^15^ and decreased neural connectivity between the prefrontal cortex (PFC) and basolateral amygdala^16^. Brain-derived neurotrophic factor (BDNF) is the most abundant neurotrophin in the central nervous system and regulates anxiety and fear extinction in the medial PFC (mPFC)^17–20^. Early-weaned mice exhibit decreased BDNF levels in the hippocampus^21^ and PFC and decreased *BDNF* exon III mRNA^11^, which may affect social cognition and emotional expression in both humans and rodents^22,23^. Therefore, BDNF signaling might be responsible for early weaning-induced neural dysfunction.

However, the neurochemical mechanisms of early weaning-induced anxiety remain unestablished. Revealing the molecular mechanisms behind it would beneficial to ameliorating mental problems caused by abuse in childhood in humans, such as anxiety, post-traumatic stress disorder and depression. To elucidate these mechanisms, we tested for a causal relation between heightened HPA axis activity and heightened anxiety behavior in early-weaned mice and aimed to identify the molecules and brain regions responsible for early weaning’s neural and behavioral effects. We hypothesized that BDNF in mPFC is involved in early weaning-induced behavioral changes.

## Results

### Inhibition of PFC glucocorticoid action in adulthood ameliorated anxiety in early-weaned mice

**Figure 1:**
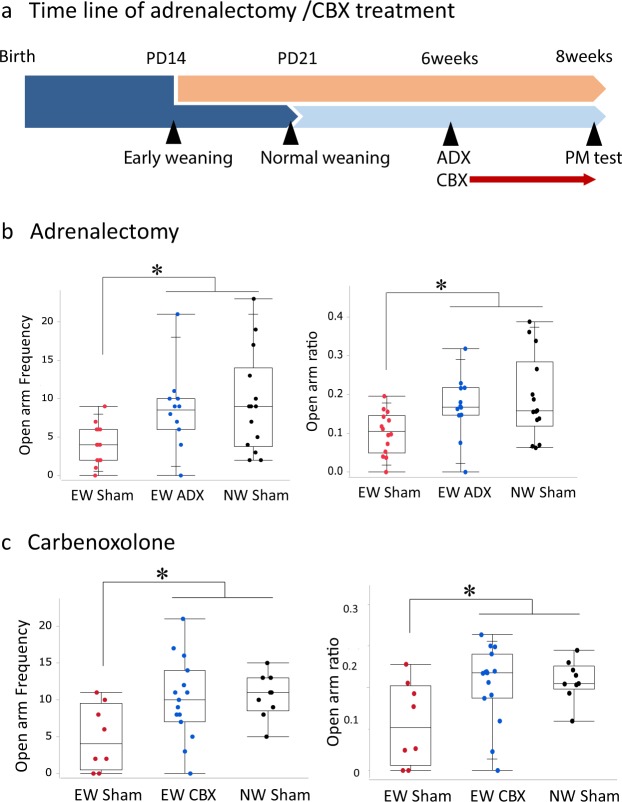
Effects of circulating corticosterone on anxiety in early-weaned mice in the adulthood. (**a**) The timeline of this experiment, the early- and normally weaned mice were adrenoectomized or treated with carbenoxolone (CBX) from PD42 and their behavior was tested on PD56. The following panels depict different groups’ open arm entry frequencies and ratios of open arm entries to total arm entries. (**b**) Comparison of adrenalectomized early-weaned mice on PD42-44 (n = 12), sham-operated early-weaned mice (n = 14), and sham-operated normally weaned mice (n = 14). (**c**) Comparison of carbenoxolone-treated early-weaned mice (n = 15), vehicle-treated early-weaned mice (n = 8), and vehicle-treated normally weaned mice (n = 9).

As adults, early-weaned mice show higher circulating corticosterone levels than normally weaned mice^13^. To determine whether elevated circulating corticosterone caused increased anxiety in the early-weaned mice, adrenalectomies were conducted on postnatal days 42–44 (PD42-44), and their behaviors were measured on PD56 (Fig. 1a). The adrenalectomized early-weaned mice exhibited open arm entry frequencies and ratios of open arm entries to total arm entries (hereafter referred to as open arm ratios) greater than those of the sham operated early-weaned mice and comparable to those of the sham operated normally weaned mice (Fig. 1b). Open arm frequency as well as the ratio of open arm frequency to total entry was different among the groups (Open arm frequency; Kruskal–Wallis test, χ^2^ = 9.14, p < 0.05. Open arm ratio; Kruskal–Wallis test, χ^2^ = 8.65, p < 0.05.), and the early-weaned sham group was lower than the other two groups (Mann-whitney *U* test with Bonferroni correction, p < 0.05).

Inhibiting corticosterone secretion by oral administration of the 11β-hydroxysteroid dehydrogenase inhibitor carbenoxolone (10 mg/kg) from PD42 to PD56 also ameliorated anxiety-related behavior in the early-weaned mice (Fig. 1a). Open arm frequency as well as the ratio of open arm frequency to total entry was different among the groups (Fig. 1c. Open arm frequency; Kruskal–Wallis test, χ^2^ = 6.49, p < 0.05. Open arm ratio; Kruskal–Wallis test, χ^2^ = 6.25, p < 0.05.), and the early-weaned sham group was lower than the other two groups (Mann-whitney *U* test with Bonferroni correction, p < 0.05). These results indicate that elevated adrenal corticosterone secretion contributes to elevated anxiety in the early-weaned mice.

### Corticosterone acts on the PFC and elevates anxiety

**Figure 2:**
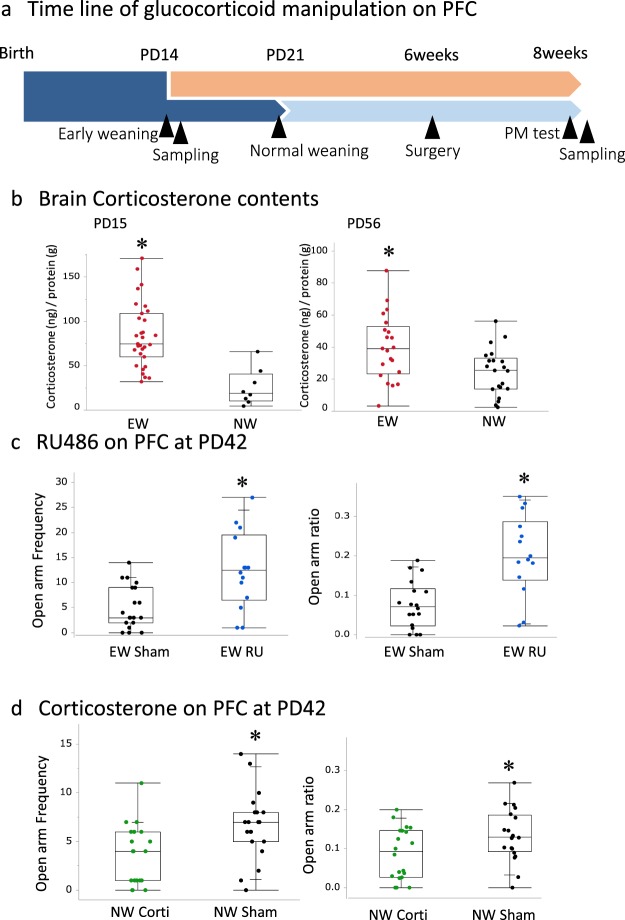
Effects of PFC corticosterone activity in adulthood on anxiety in early-weaned mice. (**a**) The timeline of this experiment, the early- and normally weaned mice were implanted with RU486, a glucocorticoid receptor antagonist, or corticosterone, respectively, from PD42 and their behavior was tested on PD56. (**b**) Corticosterone concentrations on PD15 and PD56. PFC regions were punched out and corticosterone concentrations were measured by ELISA (ng/protein(g)). The following panels depict different groups’ open arm entry frequencies and ratios of open arm entries to total arm entries. (**c**) Early-weaned mice treated with PFC implants of RU486 (n = 14) are compared to sham-operated early-weaned mice (n = 19). (**d**) Normally weaned mice treated with PFC implants of corticosterone (n = 20) are compared with sham-operated normally weaned mice (n = 20).

In identifying the brain region responsible for early weaning-induced anxiety, we considered the PFC a candidate because BDNF expression is correlated with fear-related memory in early-weaned mice^11^. This indicates that early-weaned mice have greater PFC corticosterone activity than normally weaned mice do. Indeed, the early-weaned mice had greater PFC corticosterone levels than normally weaned mice on PD15 and PD56 (Fig. 2b: PD15, z = −3.84, p < 0.0001. PD56 z = −2.59, p < 0.01. Mann-whitney *U* test).

Glucocorticoid signaling was pharmacologically manipulated with chronic diffusion from Elvax PFC implants on PD42 (Fig. 2a). Compared to sham-operated early-weaned mice, early-weaned mice that received the glucocorticoid receptor (GR) antagonist RU486 on PD 42-44 and were placed in the elevated plus maze on PD56-58 exhibited higher open arm entry frequencies and open arm ratios (Fig. 2c. Open arm frequency; Mann-whitenny test, z = 2.84, p < 0.01. Open arm ratio; Mann-whitenny test, z = 3.11, p < 0.005).

Moreover, compared to sham-operated normally weaned mice, normally weaned mice that received corticosterone on PD 42-43 and were placed in the elevated plus maze on PD56-58 exhibited lower open arm entry frequencies and open arm ratios (Fig. 2d. Open arm frequency; Mann-whitenny test, z = 2.89, p < 0.01. Open arm ratio; Mann-whitenny test, z = 1.88, p < 0.05). These results indicate that elevated PFC corticosterone activity contributes to early weaning-induced anxiety.

### Prolonged corticosterone secretion in pups after early weaning acts on the PFC and induces higher anxiety in adulthood

**Figure 3:**
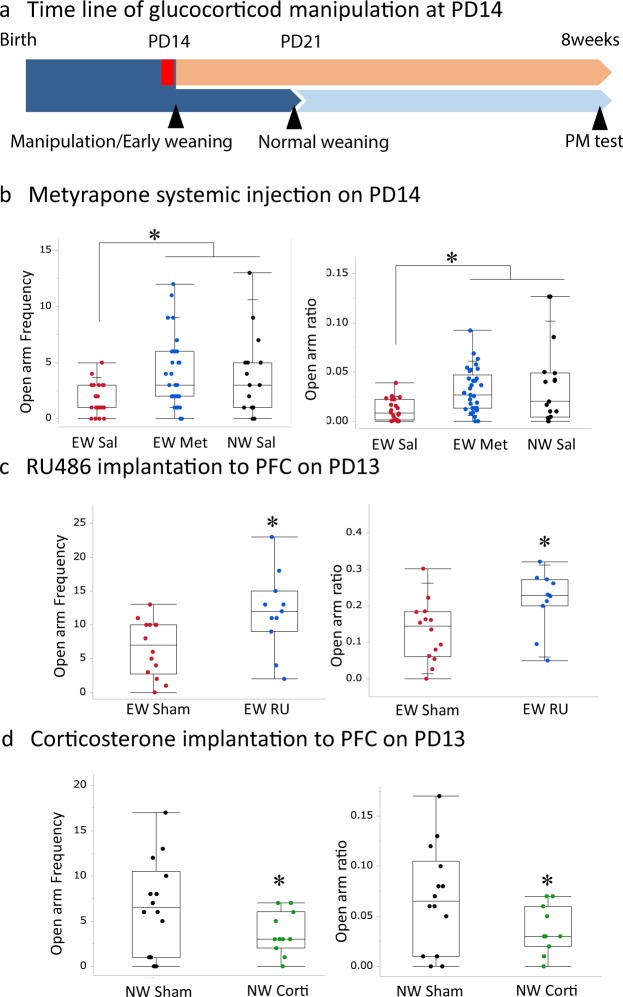
Effects of enhanced corticosterone activity immediately after weaning on anxiety in early-weaned mice. (**a**) The timeline of this experiment, the early- and normally weaned mice were treated with metyrapone, or RU486, a glucocorticoid receptor antagonist, or corticosterone, respectively, from PD13 and their behavior was tested on PD56-58. The following panels depict different groups’ open arm entry frequencies and ratios of open arm entries to total arm entries. (**b**) Systemic metyrapone-treated early-weaned mice are compared to saline-treated early-weaned mice. (**c**) Early-weaned mice treated with PFC implants of RU486 (n = 11) are compared to sham-operated early-weaned mice (n = 14). (**d**) Normally weaned mice treated with PFC implants of corticosterone (n = 14) are compared to sham-operated normally weaned mice (n = 14).

Early-weaning increases corticosterone secretion for over 48 hours after PD14 weaning^21^, so this secretion could mediate early weaning-induced anxiety. Therefore, the effects of increase corticosterone immediately after the weaning was manipulated (Fig. 3a). When early-weaned pups were treated with metyrapone (50 mg/kg in saline), an 11β-hydroxylase inhibitor that blocks corticosterone synthesis, every 4 hours from immediately before weaning to 48 hours later, they exhibited corticosterone secretion equal to that of pups that stayed with the dam (Supplementary Fig. S1. Mann-whitenny test, z = 2.08 (with Dam vs Separated), z = 2.32 (Met vs Sal), p < 0.05). In adulthood, the metyrapone-treated pups exhibited anxiety-related behaviors no greater than those of normally weaned, saline-treated mice (Fig. 3b). Open arm frequency as well as the ratio of open arm frequency to total entry was different among the groups (Open arm frequency; Kruskal–Wallis test, χ^2^ = 8.22, p < 0.05. Open arm ratio; Kruskal–Wallis test, χ^2^ = 10.27, p < 0.01). The early-weaned saline-treated group showed lower open arm entry frequencies and open arm ratios as compared to other two groups (Mann-whitney *U* test with Bonferroni correction, *p < 0.05).

To reveal the brain target of circulating corticosterone in the early-weaned pups, we pharmacologically manipulated glucocorticoid signaling using Elvax PFC implants (Fig. 3a). Compared to sham-operated early-weaned mice, early-weaned mice that received RU486 on PD13 and were placed in the elevated plus maze on PD56-58 exhibited greater open arm entry frequencies and open arm ratios (Fig. 3c). Open arm frequency as well as the ratio of open arm frequency to total entry was higher in the RU486 treated groups as compared to the sham-operated one (Open arm frequency; Mann-whitenny test, z = 2.44, p < 0.05. Open arm ratio; Mann-whitenny test, z = 2.54, p < 0.05).

Moreover, compared to sham-operated normally weaned mice, normally weaned mice that received corticosterone on PD13 and were placed in the elevated plus maze on PD56-58 (Fig. 3a) exhibited lower open arm entry frequencies and open arm ratios (Fig. 3d. Open arm frequency; Mann-whitenny test, z = 1.87, p < 0.05. Open arm ratio; Mann-whitenny test, z = 1.88, p < 0.05). These results indicate that heightened PFC corticosterone activity after early weaning contributes to early weaning-induced anxiety in adulthood.

### Early-weaning decreased PFC expression of BDNF protein and BDNF promoter region mRNA

**Figure 4:**
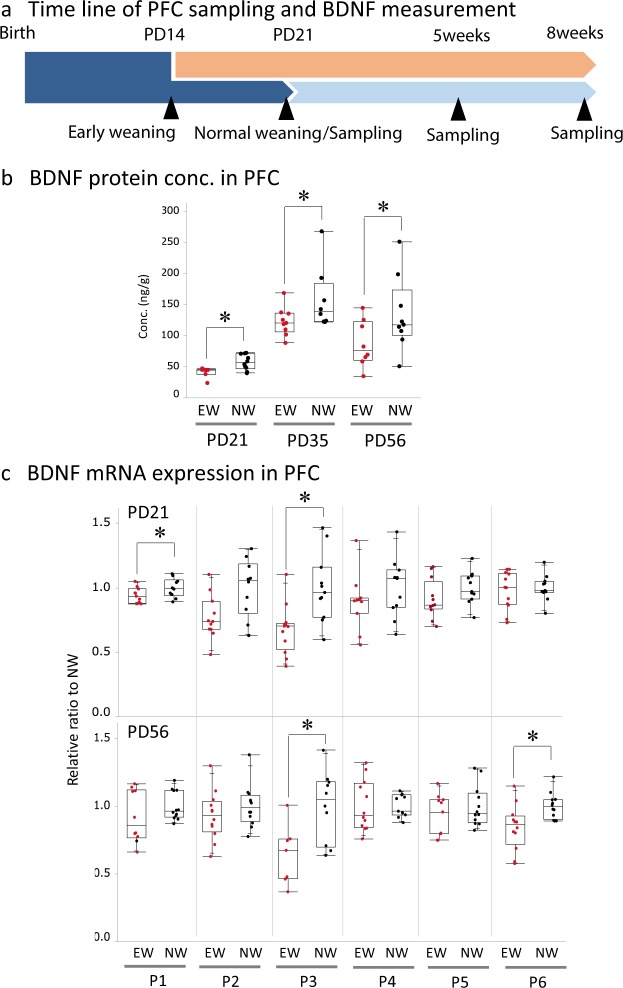
Effects of early weaning on PFC expression of BDNF. (**a**) The timeline of sampling, the brain PFC was harvested on PD 21, 35 and 56. (**b**) BDNF protein contents in PFC. (PD21, early-weaned, n = 8; normally weaned, n = 10. PD35, early-weaned, n = 9; normally weaned, n = 8. PD56, early-weaned, n = 8; normally weaned, n = 9). (**c**) The *BDNF* mRNA expression in the specific *BDNF* promoter regions (PD21, early-weaned, n = 18; normally weaned, n = 18. PD56, early-weaned, n = 18; normally weaned, n = 18) on PD21, PD35 (BDNF protein only), and PD56. Early-weaned mice are compared to normally weaned mice.

Early-weaning decreased PFC expression of BDNF and *BDNF* exon III mRNA, suggesting that elevated corticosterone might act on PFC neurons and decrease BDNF synthesis. Immunochemical analysis showed that approximately 85% of BDNF-containing neurons were positive for the anti-GR antibody (Supplementary Fig. S2), indicating that corticosterone acts on BDNF-expressing PFC neurons. The PFC was dissected on PD21, 35 and 56, and the BDNF protein levels as well as each BDNF promoter specific mRNA was measured (Fig. 4a). Compared to normally weaned mice, early-weaned mice had lower BDNF protein contents on PD21, PD35, and PD56 (Fig. 4b. F_5,46_ = 12.42, p < 0.0001, two-way ANOVA. *p < 0.05, two-tailed t-test between the early- and normally weaned groups). Regarding to promoter specific mRNA expression, compared to normally weaned mice, the early-weaned mice exhibited decreased expression of *BDNF* exon I and III mRNA on PD21 and *BDNF* exon III and VI mRNA on PD56 (Fig. 4c. *p < 0.05, two-tailed t-test). These results show that early weaning reduces the expression of BDNF and specific promoter region mRNAs, especially *BDNF* exon III.

### Enhanced BDNF signaling after early-weaning ameliorated anxiety-related behavior in adulthood

**Figure 5:**
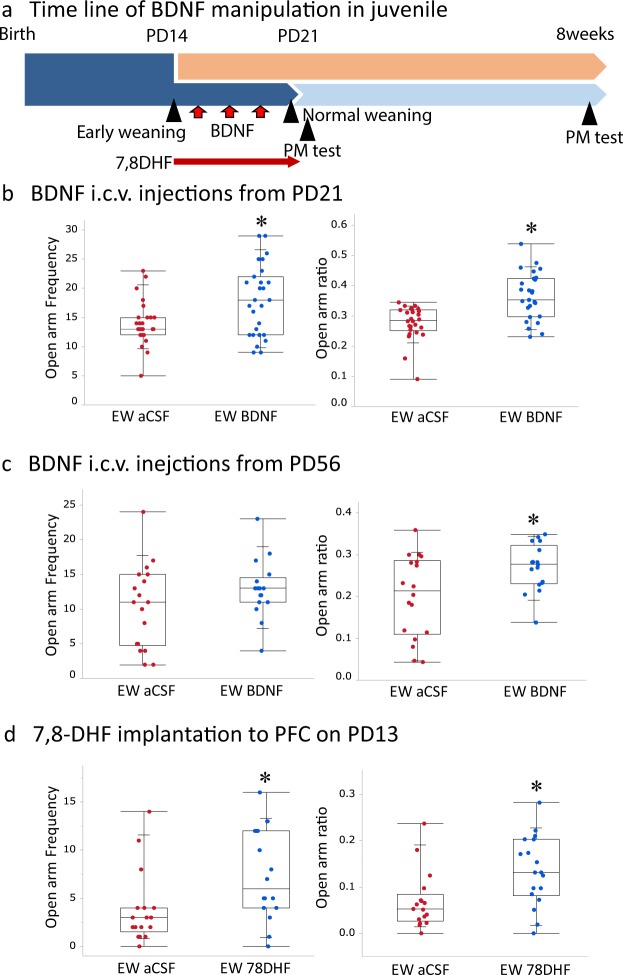
Effects of BDNF signaling on anxiety in early-weaned mice. (**a**) The timeline of this experiment, the early- and normally weaned mice were injected with BDNF (i.c.v.) from PD 15, 17 and 19, and their behavior was tested on PD21 and 56. The another set of the mice were implanted with 7,8-DHF, a trkB agonist, to PFC on PD13 and their behavior was tested on PD56. The following panels depict different groups’ open arm entry frequencies and ratios of open arm entries to total arm entries. (**b**,**c**) BDNF-treated early-weaned mice (n = 14) are compared to sham-treated early-weaned mice (n = 15). (**d**) Early-weaned mice treated with PFC implants of 7,8-DHF (n = 18) are compared to sham-treated early-weaned mice (n = 17).

To reveal the function of early weaning-induced reductions in BDNF levels, we pharmacologically manipulated BDNF signaling with intraventricular BDNF injections (Fig. 5a). Compared to vehicle-treated early-weaned mice, the BDNF-treated early-weaned mice exhibited higher open arm entry frequencies on PD21 (Mann-whitenny test, z = −2.11, p < 0.05) and greater open arm ratios on PD21 (Mann-whitenny test, z = −4.05, p < 0.0001), and on PD56 (Mann-whitenny test, z = 2.00, p < 0.05) (Fig. 5b,c). To reveal the brain region-specific function of BDNF signaling in the early-weaned pups, we pharmacologically manipulated BDNF-tyrosine receptor kinase B (trkB) signaling using Elvax PFC implants of trkB agonist 7,8-dihydroxyflavone (7,8-DHF) on PD13 (Fig. 5a). The mice were placed in the elevated plus maze on PD56, and compared to vehicle-treated early-weaned mice, early-weaned mice that received 7,8-DHF exhibited higher open arm entry frequencies and open arm ratios (Open arm frequency; Mann-whitenny test, z = 4.71, p < 0.05. Open arm ratio; Mann-whitenny test, z = 5.13, p < 0.05) (Fig. 5d). These results suggest that restoring BDNF function in juvenile early-weaned mice ameliorates anxiety in adulthood.

## Discussion

Early weaning in rodents induces persistent anxiety and heightened HPA activity^13^. We aimed to explore the neuromolecular mechanisms underlying the anxiogenic effects of early-weaning. We hypothesized that BDNF is involved in early weaning-induced behavioral changes. We regard our results as confirming our hypothesis. We found that inhibiting HPA activity by adrenalectomy or corticosterone synthesis inhibition normalized anxiety in adulthood, indicating that heightened HPA activity elevates anxiety in the early-weaned mice.

Circulating corticosterone can act on GR-expressing neurons such as the PFC neurons that might modulate anxiogenic influences during development. For example, mice that lack GR expression in the forebrain, which includes the PFC and limbic system, exhibit reduced anxiety-related behavior^24^. PFC neurons project axons to the basolateral amygdala, which controls the expression of anxiety-related behavior^25^. Moreover, early weaning increases myelination in the basolateral amygdala^15^, decreases neural connectivity between the PFC and amygdala^16^, and decreases PFC BDNF levels^11^. In this study, we found that inhibiting PFC GR signaling reduced anxiety-related behavior in the early-weaned mice. This suggests that the PFC is among the brain regions responsible for controlling anxiety, but we have not tested other brain regions such as the amygdala or hippocampus, so their GRs might also contribute to the elevated anxiety in the early-weaned mice in an orchestrated neural network.

The neurobehavioral phenotypes of the early-weaned mice originated in the post-weaning developmental period. We previously reported that early-weaned mice secreted more corticosterone at least 48 hours after weaning^21^, so prolonged corticosterone elevation might cause the persistent anxiety behaviors of the early-weaned mice. Treating early-weaned mice with metyrapone, which normalizes corticosterone secretions, ameliorated their anxiety-related behavior in adulthood. Similarly, reducing PFC corticosterone action with RU486 reduced anxiety-related behaviors. Moreover, even mouse pups that stayed with their dam exhibited heightened anxiety-related behaviors following PFC corticosterone implants. While RU486 has a potential to inhibit progesterone receptor, these results suggest that elevated HPA activity immediately after weaning heightens anxiety-related behaviors by acting on the PFC. More specific blockade of glucocorticoid receptors action using genetic knockdown of the receptor in the PFC or optogenetic manipulations are needed in future to confirm these results.

Stress in late suckling and early adolescence (PD14-59) in rodents affects brain development, particularly in the PFC^26,27^. Interestingly, prepubescent rats exhibit prolonged glucocorticoid release in response to stress^28^ because of incomplete maturation of negative feedback systems^29^. These results are consistent with previous reports that early-weaned mice exhibit prolonged corticosterone secretion for over 48 hours^21^, suggesting that immature brain regions including the PFC are severely affected by excessive corticosterone levels, causing persistent anxiety. Another interesting point is that stress in late suckling and early adolescence can be characterized by “potentiation/incubation effects,” to which the frontal cortex may be the most vulnerable region, possibly causing protracted glucocorticoid release that persists into adulthood^26^. In our first experiment, reducing the heightened glucocorticoid signaling in early-weaned adult mice reduced anxiety-related behaviors, suggesting that early weaning-induced prolongation of corticosterone secretion impairs PFC function, leading to heightened responses to novel stressors and greater anxiety-related behavior, as seen in the plus maze test.

Stress activates the HPA axis and stimulates corticosterone secretion. Chronically elevated corticosterone adversely affects the structure and function of the limbic brain regions^30^. These corticosterone-induced changes are similar to the effects of BDNF dysfunction, such as neural cell death^31,32^ and decreased neurogenesis^33,34^. Circulating corticosterone might therefore act on BDNF signaling and affect neural functions^35^.

Corticosterone treatment decreases BDNF mRNA and protein levels^36^. Interestingly, this inhibitory effect of corticosterone is enhanced following earlier corticosterone exposure^37^. This is consistent with early-weaned mice exhibiting heightened corticosterone on PD14-16^21^ and then exhibiting it again after a mild stress^13^, suggesting that repeated corticosterone exposure inhibits BDNF synthesis. A study of mice that underwent a fear-conditioning and fear-extinction procedure found that early-weaned mice exhibited lower BDNF synthesis than normally weaned mice even one month after the behavioral test^11^, suggesting that foot-shock-induced corticosterone secretion deepens the inhibition of BDNF synthesis in early-weaned mice.

In rodents, several *BDNF* exons have been identified^38^. The precise mechanism for the inhibition of specific *BDNF* promoters is unknown, it is hypothesized that corticosterone can alter the transcription of exons II and VI^39,40^, but early-weaned mice exhibit lower mPFC expression levels of BDNF and *BDNF* exon III transcripts than normally weaned mice do^11^. These levels were negatively correlated with fear responses observed during extinction training, suggesting that early-weaned mice had some inhibition of *BDNF* exon III transcripts and consequent fear-related behaviors. Tsankova *et al*. showed that social stress inhibited *BDNF* transcripts III and IV through chromatin remodeling^40^, and we had similar results of inhibition of *BDNF* III. Corticosterone not only inhibits *BDNF* transcription but also alters the stability, translation, and allocation of mRNAs^35^. The detailed molecular mechanisms of PFC corticosterone-BDNF interactions will need to be clarified in future studies.

BDNF can be decreased in abused children, and chronic stress in rats increases anxiety and depressive-like behaviors, similar to early weaning^41–43^. These rodents exhibit reduced PFC GR expression and impaired BDNF function^19^. Maternally deprived rats exhibit lower PFC BDNF levels and higher serum corticosterone levels than non-deprived rats^44^. These deprived rats also exhibit elevated anxiety-related behavior. Consistent with these reports, we found that treating early-weaned mice with i.c.v.-administered BDNF or the PFC-TrkB agonist 7,8-DHF on PD13 ameliorated anxiety behaviors. PFC neurons project axons to the basolateral amygdala, which a regulatory center for anxiety^45^. Additionally, neural connectivity between the PFC and basolateral amygdala is impaired in early-weaned rats^16^. This suggests that early weaning-induced corticosterone secretion inhibits PFC BDNF signaling and decreases PFC regulation of the basolateral amygdala, resulting in increased anxiety. Further studies are needed to clarify the causal relationship between dysfunctional PFC-basolateral amygdala connections and elevated anxiety in early-weaned mice.

In conclusion, early weaning disrupts the mother-infant bond and leads to increased corticosterone secretion, higher anxiety, and elevated stress responses in adulthood. We found that 1) normalizing PFC corticosterone signaling in the early-weaned mice ameliorated their anxiety in the plus maze test, indicating that their anxiety-related behavior is caused by heightened HPA activity affecting the PFC, 2) corticosterone was the molecule mediating early weaning’s effects, 3) early-weaned mice exhibited prolonged depression of PFC BDNF synthesis and *BDNF* exon III mRNA levels. Finally, we found that 4) pharmacologically activating PFC BDNF signaling ameliorated anxiety in the early-weaned mice. Collectively, these findings indicate that disruption of mother-infant bonding increases anxiety via modulation of PFC glucocorticoid-BDNF signaling. The developing brain is vulnerable to stress and shaped by the social environment, and these adolescent social experiences are encoded in the brain and have enduring behavioral effects. Understanding the neural mechanisms responsible for elevated anxiety and stress responses originating in infancy would have clinical benefits, especially in treating abused children.

## Materials and Methods

The protocol of the animal research was approved by Ethical committee of Azabu University (#160303-5) and carried out in accordance with the Guidelines for Animal Experiments of Azabu University.

### Animals

We used ICR mice (Japan Clea, Yokohama, Japan) in all experiments. We followed a published weaning protocol^9,10,12^. Briefly, when female mice became pregnant, they were checked each morning until parturition. For each litter, the date of birth was designated PD0. On PD2, each litter was culled to ten pups, with five pups of each sex. Throughout the nursing period, we avoided disturbing the animals except for a brief weekly cage cleaning. On PD14, half the litter of mixed sex was separated from each dam, assigned to the early-weaned group, and fed powdered pellets until the remaining pups were weaned on PD21 (normally weaned group).

### Elevated plus-maze test

We used a standard plus maze apparatus (25 × 5 cm closed arm with a 5 cm-high wall) located 20 cm above the floor^9,10,12^. Each animal was placed in the neutral zone facing the open arm, and its behavior was filmed for 15 min, and the entry to the open arms was used for group comparison. The procedure and data analysis were identical to those of previous studies^9,10,12^.

### Surgeries

On PD42-44, mice were underwent to adrenalectomy. A small incision was made on the lateral abdomen, and the adrenal grand was dissected by a small scissor under the isoflurane anesthesia (2%). Both sides of the adrenal were dissected.

To chronically modulate specific PFC neurochemical pathways, we surgically implanted drug delivery systems in relevant brain regions on PD13 or PD42, under the isoflurane anesthesia (0.5%)^46,47^. Corticosterone, RU486, or the BDNF-TrkB agonist 7,8-DHF^48^ were locally administered into the PFC by continuous infusion from Elvax implants for over a week^46,47^. The control group of each experiment was mice that received Elvax implants that weren’t loaded with any drug. The detail methods for preparation of the drug-containing Elvax was described in Supplementary materials.

### Intracranial injection

Mouse pups were anesthetized with isoflurane (2–5%), and a 25-gauge needle was inserted into the lateral ventricle. One μl of BDNF in aCSF (0.5 μg/μl) or aCSF vehicle was intraventricularly infused. This was done on PD15, PD17, and PD19. After the injection, the mice were returned to their cages.

### Quantitative Real-Time PCR

The PFC was then isolated by coring the cortical lamina region between the forceps minor and the corpus callosum according to a mouse brain atlas using a sample corer (ID 2 mm, Fine Science Tools, Foster City, CA). The dissected tissue was primarily composed of the infralimbic mPFC. The tissue samples were stored at −80 °C in either tubes containing RNAlater RNA stabilization reagents for quantitative real-time PCR. Total RNA was extracted using the Rneasy Protect Mini Kit (Qiagen) according to the manufacturer’s protocol, followed by standard Dnase treatment using recombinant Dnase I. Real-time quantitative PCR was performed as our previous studies^11,21^.

### BDNF ELISA

Each mouse’s mPFC was homogenized in lysis buffer and centrifuged at 15,000 × *g* for 15 min at 4 °C. The supernatants were collected, and their protein concentrations were measured with a Bio-Rad protein assay kit (Bio-Rad Laboratories). BDNF levels were measured using ELISA (BDNF Emax Immunoassay kit; Promega, Madison, WI) according to the manufacturer’s instructions.

### Statistical analysis

Statistical analysis was performed using JMP software (Version 12, SAS Institute, Cary, NC). We defined statistical significance as p < 0.05. For between-group behavior comparisons, Kruskal-Wallis non-parametric test, followed by the Mann-Whitney test with Bonferroni correction, were performed. Between-group comparisons of BDNF exon expression levels were performed using Levene’s test, followed by Welch’s t test. BDNF protein expression levels were compared using Student’s t test.

### Declarations

All the authors agree to publish the results of this study. The datasets used and/or analysed during the current study are available from the corresponding author on reasonable request. The authors do not have a conflict of interest in this manuscript., and this work was funded by grants from the JSPS KAKENHI (15H02479, 25118007, 23248049). TK, NK, MO, NH, KI, MT, NT, KM, HA, MY conducted the experiments, TK, MN, and KM planed the experiments. TK and KM wrote the paper. MN conducted the statistical analysis. The authors thank Dr. Yuko Shimokawa, Ms. Misato Takaki, Tomoko Hatanaka, Yuiko Ishida, Yukino Ishio, Sinsuke Moride, Akihide Nakamura, Ayu Aratame and Yuka Honda for helping the experiments.

## Supplementary information

Supplementary material
