# Supplementary material for: Early weaning increases anxiety via brain-derived neurotrophic factor signaling in the mouse prefrontal cortex
Source: Sci Rep. 2019 Mar 8;9:3991. doi: 10.1038/s41598-019-40530-9 (PMC6408497; doi:10.1038/s41598-019-40530-9)
Supplement: Supplementary file 1 — Supplementary material [file 41598_2019_40530_MOESM1_ESM.doc]

Early weaning increases anxiety via brain-derived neurotrophic factor signaling in the mouse prefrontal cortex

Takefumi Kikusui, Natsumi Kanbara, Mariya Ozaki, Nozomi Hirayama, Kumiko Ida, Mika Tokita, Naho Tanabe, Kuriko Mitsuyama, Hatsuki Abe, Miki Yoshida, Miho Nagasawa and Kazutaka Mogi*

Companion Animal Research, School of Veterinary Medicine, Azabu University, Sagamihara 252-5201, Japan

* Correspondence to Kazutaka Mogi, D.V.M., Ph.D. Companion Animal Research, School of Veterinary Medicine, Azabu University, Sagamihara 252-5201, Japan

Tel: +81-42-769-1853 Fax: +81-42-850-2513

E-mail: [mogik@azabu-u.ac.jp](mailto:mogik@azabu-u.ac.jp)

**Methods**

*Animals*

We used ICR mice (Japan Clea, Yokohama, Japan) in all experiments. All animal experiments were approved by the Azabu University Ethics Committee (#160303-5). Male and female mice were paired in cages (175 × 245 × 125 mm) for breeding. Food and water were supplied ad libitum. The environment was maintained at 24 ± 1°C and 50 ± 5% humidity under a 12-h light–dark cycle (lights on at 0800).

*Antibodies and reagents*

We obtained corticosterone from Wako Chemicals, Co. Ltd., RU486 from Abcam Plc. (ab120356), 7,8-DHF from Abcam Plc. (ab120996), Elvax beads from DuPont (Wilmington, DE), and BDNF from Abcam Plc. (ab9794). We used an artificial cerebrospinal fluid (aCSF) containing NaCl, 127 mM; NaHCO3, 26 mM; KCl, 1.5 mM; KH2PO4, 1.24; MgSO4, 1.4 mM; CaCl2, 2.4 mM; Glucose, 10 mM. We obtained Triton X-100 from Wako Chemicals, Co. Ltd. And added 0.3% Triton X-100 to 0.1 M PBS to create our wash solution (used for all wash steps unless otherwise specified).

For processing mPFC tissues, we used RNAlater RNA stabilization reagents (Qiagen, Venlo, Netherlands) and a pH 8.0 lysis buffer containing 20 mM Tris-HCl, 137 mM NaCl, 1% NP-40, 10% glycerol, 1 mM phenylmethylsulfonyl fluoride, 10 μg/ml aprotinin, 1 μg/ml leupeptin, and 0.5 mM NaVO_3_. When isolating RNA, we used recombinant Dnase I from Takara Bio (Shiga, Japan). For polymerase chain reaction (PCR), we used SYBR Premix Ex Taq II from Takara Bio.

*Weaning procedures*

We followed a published weaning protocol (9, 10, 12). Briefly, when female mice became pregnant, they were checked each morning until parturition. For each litter, the date of birth was designated PD0. On PD2, each litter was culled to ten pups, with five pups of each sex. Throughout the nursing period, we avoided disturbing the animals except for a brief weekly cage cleaning. On PD14, half the litter of mixed sex was separated from each dam, assigned to the early-weaned group, and fed powdered pellets until the remaining pups were weaned on PD21 (normally weaned group). Thereafter, both groups were fed pellets. After weaning, two or three littermate pups were housed together according to weaning group and sex. The littermates were randomly assigned to one of three treatments, the early-weaned control, normally weaned control and experimental group, to avoid litter effects, and male pups were used in the following experiments because males are more vulnerable to early-weaning stress (6, 7). This early-weaning manipulation caused a minimum body weight loss in the pups, but the effects were not reached to the significant level (Supplementary Table S1).

*Elevated plus-maze test*

The elevated plus-maze test was conducted during the light–dark cycle’s light period under a dim red light with the room lights turned off. We used a standard plus maze apparatus (25 × 5 cm closed arm with a 5 cm-high wall) located 20 cm above the floor (9, 10, 12). Each animal was placed in the neutral zone facing the open arm, and its behavior was filmed for 15 min. We measured the frequency of entering the arms, dwell time in the arms, latency to enter the arms, and total locomotion time. The procedure and data analysis were identical to those of previous studies (9, 10, 12). The entry of the open arms was more sensitive to the effects of the early-weaning and to make it consistent with past reports, we selected these open arm parameters for comparisons (9, 10, 12)”.

*Surgical drug administration.*

To chronically modulate specific PFC neurochemical pathways, we surgically implanted drug delivery systems in relevant brain regions (46, 47). Corticosterone, RU486, or the BDNF-TrkB agonist 7,8-DHF (48) were locally administered into the PFC by continuous infusion from Elvax implants for over a week (46, 47). The control group of each experiment was mice that received Elvax implants that weren’t loaded with any drug. Elvax beads were dissolved in dichloromethane and mixed with dimethyl sulfoxide (DMSO) containing Fast green FCF and a solution containing the chosen drug (46, 47). After homogenization by stirring, the solution was frozen quickly, kept at -80°C for 1 h, and then stored at -20°C overnight to allow the dichloromethane to evaporate. The final concentrations of Elvax, Fast green FCF, and the chosen drug were 2.5 mg/ml, 1%, and 0.5 M, respectively, in a solution that was 50% DMSO. For the Elvax piece implantation, PD13 or PD42-44 mice were anesthetized with isoflurane (2-5%), the skin over the PFC was cut, and a small craniotomy was performed over the PFC. A piece of Elvax Sheet (4 × 4 mm) was placed in the PFC cerebral fissures, the hole was then covered with the piece of bone with surgical bond, and the skin was sutured. The final drug amount in the Elvax piece was 250 mmol for each drug.

*mPFC dissection*

Mice were euthanized by cervical dislocation. Their brains were immediately removed, and a 1-mm slice was coronally cut approximately 1.3-2.3 mm anterior from the bregma using a brain slicer (MK-MC-01, Muromachi Kikai, Tokyo, Japan) on ice. The PFC was then isolated by coring the cortical lamina region between the forceps minor and the corpus callosum according to a mouse brain atlas (50) using a sample corer (ID 2 mm, Fine Science Tools, Foster City, CA). The dissected tissue was primarily composed of the infralimbic mPFC. The tissue samples were stored at -80 °C in either tubes containing RNAlater RNA stabilization reagents for quantitative real-time PCR or tubes containing lysis buffer for enzyme-linked immunosorbent assays (ELISAs).

*Isolation of RNA and reverse transcription*

Total RNA was extracted using the Rneasy Protect Mini Kit (Qiagen) according to the manufacturer’s protocol, followed by standard Dnase treatment using recombinant Dnase I. The concentration and integrity of the final eluates were determined with a NanoDrop 2000 (Thermo-Fisher). For reverse transcription, we selected samples with 260 to 280 nm absorbance ratios greater than 1.5. For each sample, 2 ng of total RNA was reverse transcribed into cDNA using the SuperScript VILO cDNA Synthesis Kit (Life Technologies, Carlsbad, CA) on a thermal cycler (MyCycler, Bio-Rad Laboratories, Hercules, CA) at 25 °C for 10 min, 42 °C for 60 min, and 85 °C for 5 min. The cDNA products were stored at -20 °C before real-time PCR amplification.

*Quantitative Real-Time PCR*

Real-time quantitative PCR was performed with a Thermal Cycler Dice Real Time System Single TP850 (Takara Bio) using the SYBR Premix Ex Taq II according to the manufacturer’s instructions. PCR primer sequences were designed to distinguish *BDNF* exons I, II, III, IV, V, and IX and hypoxanthine phosphoribosyltransferase (*HPRT*), which was used as the reference gene ^11^. The primer designs were based on GenBank sequences. *BDNF* exon I was amplified at 95°C for 30 s, followed by 40 cycles of 95 °C for 5 s and 55 °C for 45 s. *BDNF* exons II, IV, V, and IX and *HPRT* were amplified at 95 °C for 30 s, followed by 40 cycles of 95 °C for 5 s and 60 °C for 45 s. *BDNF* exon III was amplified at 95 °C for 30 s, followed by 40 cycles of 95 °C for 5 s and 60 °C for 30 s. All samples were run in triplicate, and each amplification’s specificity was confirmed by analyzing the corresponding dissociation curve. The target gene expression levels were normalized to *HPRT* expression using the standard curve method. To account for litter effects in our statistical analyses, relative gene expressions were standardized by the average value of normally weaned littermates.

*BDNF ELISA*

Each mouse’s mPFC was homogenized in lysis buffer and centrifuged at 15,000 × *g* for 15 min at 4 °C. The supernatants were collected, and their protein concentrations were measured with a Bio-Rad protein assay kit (Bio-Rad Laboratories). BDNF levels were measured using ELISA (BDNF Emax Immunoassay kit; Promega, Madison, WI) according to the manufacturer’s instructions. Each sample’s BDNF signal was normalized to the total protein signal. To control for litter effects, we compared relative protein expressions between weaning groups by standardizing them to the average expression in normally weaned littermates.

*Immunohistochemistry*

For immunohistochemistry, we used normal goat serum (NGS; S-100, Vector Laboratories, Burlingame, CA), anti-BDNF antibody (N20, Santa Cruz Biotechnology, Dallas, TX), biotinylated goat anti-rabbit immunoglobulin G (anti-rabbit IgG; BA-100, Vector Laboratories), anti-GR antibody (M-20, Santa Cruz Biotechnology), and anti-rabbit IgG Alexa 594 (R37119, Thermo-Fisher, Waltham, MA). We used a peroxidase substrate solution (0.05% 3,3’-diaminobenzidine, 0.015% H_2_O_2_, and 0.05% NiCl_2_ in 0.175 M NaCH_3_CO_2_ buffer). We also used Fluoromount #0245 mounting medium (Cosmo-Bio, Tokyo, Japan).

Each brain was coronally cut into 30-μm sections using a cryostat. Tissue sections were washed with 0.1 M PBS and treated with 0.6% H_2_O_2_ in methanol for 30 min at room temperature (RT). The sections were rinsed with wash solution, incubated with 5% NGS in wash solution for 1 h at RT, and incubated with anti-BDNF antibody (1:500) and 5% NGS in wash solution for 48 h at 4°C. After incubation, the sections were washed, incubated again with anti-rabbit IgG (1:1000) and 5% NGS in wash solution overnight at 4°C, and washed thrice more. The stained sections were intensified and visualized using a VECTASTAIN Elite ABC kit (Vector Laboratories) with peroxidase substrate solution. The sections were rinsed with 0.175 M NaCH_3_CO_2_ buffer for 10 min and five times with 0.1 M PBST for 5 min to remove the first antibody’s residues. The sections were then incubated with anti-GR antibody (1:100) and 5% NGS in wash solution overnight at 4°C. The sections were then washed and incubated with anti-rabbit IgG Alexa 594 (1:100) for 1 h at RT, followed by a series of three washes. The sections were then washed with water, mounted on gelatin-coated glass slides, dehydrated, and cover-slipped with Fluoromount #0245 mounting medium. Every third section was visualized and analyzed with a light microscope (BX51-N and DP71, Olympus, Tokyo, Japan). The PFC’s area was confirmed using a brain atlas(50). Example immunostained cells are shown in Supplementary Figure S1.

*Statistical analysis*

Statistical analysis was performed using JMP software (Version 12, SAS Institute, Cary, NC). We defined statistical significance as p < 0.05. For between-group behavior comparisons, the data did not fit normal distribution and we used Kruskal-Wallis non-parametric test, followed by the Mann-Whitney test with Bonferroni correction. Between-group comparisons of BDNF exon expression levels were performed using Levene’s test and the data were not equally distributed, therefore, we performed Welch’s t test which can be adapted even if the distribution of the data was not equal. BDNF protein expression levels were compared using Levene’s test, and the data were equally distributed, so Student’s t test was performed for the group comparison.

*Data availability*

The datasets generated during and/or analysed during the current study are available from the corresponding author on reasonable request.

Supplementary Table S1

|  | Early | | |  | Normal | | | *p* |
| --- | --- | --- | --- | --- | --- | --- | --- | --- |
| PD15 | 10.0 | ± | 0.57 |  | 10.4 | ± | 0.35 | 0.56 |
| PD21 | 17.7 | ± | 1.05 |  | 18.3 | ± | 0.59 | 0.60 |

(grams)

Supremely Fig.S1 Corticosterone concentrations on PD15, 24 hours after weaning. Compared to the pups stayed with dam (n=3), separation from the mother (n=5) increased corticosterone. Metyrapone-treated maternally separated pups (Met. n=5) showed lower corticosterone as compared to the saline treated (Sal) pups (n=4).

Supremely Fig.S2 Double immunostaining against BDNF (A) and BDNF (B) in mouse PFC. BDNF was in dark-blue, and the GR was in red fluorescence. Approximately 85% of BDNF positive neurons are expressing GR (C).
